# Oat beta-glucan ameliorates insulin resistance in mice fed on high-fat and high-fructose diet

**DOI:** 10.3402/fnr.v57i0.22754

**Published:** 2013-12-20

**Authors:** Jie Zheng, Nanhui Shen, Shuanghui Wang, Guohua Zhao

**Affiliations:** 1 College of Food Science, Southwest University, Chongqing, China; 2 Department of Chemistry and Chemical Engineering, Chongqing University, Chongqing, China

**Keywords:** oat beta-glucan, high-fat, high-fructose, insulin resistance, mice

## Abstract

**Methods:**

This study sought to evaluate the impact of oat beta-glucan on insulin resistance in mice fed on high-fat and high-fructose diet with fructose (10%, w/v) added in drinking water for 10 weeks.

**Results:**

The results showed that supplementation with oat beta-glucan could significantly reduce the insulin resistance both in low-dose (200 mg/kg^−1^ body weight) and high-dose (500 mg/kg^−1^ body weight) groups, but the high-dose group showed a more significant improvement in insulin resistance (*P*<0.01) compared with model control (MC) group along with significant improvement in hepatic glycogen level, oral glucose, and insulin tolerance. Moreover, hepatic glucokinase activity was markedly enhanced both in low-dose and high-dose groups compared with that of MC group (*P*<0.05).

**Conclusion:**

These results suggested that supplementation of oat beta-glucan alleviated insulin resistance and the effect was dose dependent.

Insulin resistance, a metabolic syndrome, was pronounced in type 2 diabetes patients (1–3). An accumulation of evidence showed that insulin resistance was associated with obesity, hypertension, dyslipidemia, glucose intolerance (4–6), and cardiovascular disease (7). Although several drugs are available, side effects and drug resistance are of great concern. A great number of people are seeking natural products or dietary interventions for an alternative.

Oat, a worldwide cultivated crop, is rich in the soluble dietary fiber beta-glucan which is a nonstarch polysaccharide composed of linear chains of glucose with a beta-1–3) and beta-1–4) mixed linkage structure (8). The Food and Drug Administration (FDA) recommends daily consumption of 3 g beta-glucan either from oats or barley to prevent cardiovascular diseases (9, 10). Consumption of soluble dietary fibers has been associated with lower postprandial glucose and insulin response (11). It has been shown that a significantly decreased level of fasting blood glucose and a tendency to reduce fasting blood glucose and insulin resistance in type 2 diabetic subjects was observed with oat beta-glucan (3 g/day) enriched bread for a 3-week intervention period (12). Some similar studies reported that ingestion of foods naturally high in oat or barley beta-glucan improved glycemic and insulin response in overweight or type 2 diabetes subjects (13–19). Considering the health benefits of beta-glucan, many researchers have incorporated barley or oat beta-glucan into various kinds of commonly consumed food products, including breakfast cereals, milk drinks, bread, beverages, and infant foods (20–25).

The purpose of the present study was to evaluate the effect of oat beta-glucan in improving insulin resistance in Institute of Cancer Research (ICR) mice fed on a high-fat and high-fructose diet with fructose added in drinking water. The relationship between different oat beta-glucan doses and the effect in improving insulin resistance were also evaluated.

## Materials and methods

### Materials

The content of beta-glucan from oat flour (Yikang Biotechnology Co., Ltd., Hebei, China) was determined by a streamlined enzymic method (26) using the mixed linkage beta-glucan assay kit (Megazyme, Wicklow, Ireland). The results were means of triplicate measurements, which gave values of 82.09%.

Metformin was purchased from Qidu Pharmaceutical Group Co., Ltd., (Shandong, China). Other chemicals were of analytical reagent grade.

### Animals and designing

Male ICR 6-week-old mice were obtained from the Laboratory Animal Center of Daping Hospital (Chongqing, China). They were acclimatized at a controlled temperature of 20–24°C and a relative humidity of 50–70% with a 12 h light–dark cycle for 1 week. The standard diet (Laboratory Animal Center of Daping Hospital, Chongqing, China) and water were supplied ad libitum. The composition of the standard diet is shown in Table 1. All procedures using animals obtained the approval of the Animal Experiment Committee of China, and the experiment was carried out in compliance with the Guidelines for Animal Experimentation of the biological and pharmacological research laboratories.

**Table 1:** Composition of standard diet (unit:% of diet)

Component	Standard diet
Corn flour	35
Wheat flour	15
Soybean	5
Soybean meal	15
Wheat bran	15
Sesame	6.5
Milk powder	2
Bone powder	2.5
Yeast powder	2
Salt	0.5
Vitamin mixture	0.5
Rapeseed oil	1
Total (%)	100

After 1 week of acclimation, the ICR mice were randomly divided into five dietary groups (*n*=10 for each group), including normal control group (NC), model control group (MC), positive control group (PC) as well as low-dose and high-dose oat beta-glucan control groups (referred to as low-dose and high-dose groups, respectively). The NC mice were fed with the standard diet and tap water; the other four groups were fed with a high-fat and high-fructose diet along with fructose (10%, w/v) added in drinking water. Meanwhile, the low-dose, high-dose, and PC groups of mice were orally administered with either 200 mg/kg^−1^ body weight or 500 mg/kg^−1^ body weight of oat beta-glucan, or 500 mg/kg^−1^ body weight of metformin per day for 10 weeks. All the mice had free access to food and water throughout the experimental period, and their body weights were recorded weekly. The high-fat and high-fructose diet consisted of 10% lard, 10% fructose, 1% cholesterol, 0.25% pig bile salt, and 78.75% standard diet.

### Oral glucose tolerance test

Oral glucose tolerance test (OGTT) was conducted after completion of 6 weeks of the experiment. After a 12 h fasting, the mice were orally gavaged with glucose (2 g/kg^−1^ body weight), and blood glucose concentrations were measured using blood samples taken from the tail vein at 0, 30, 60, and 120 min after glucose administration with an Accu-Chek Active blood glucose meter (Roche Diagnostics, Mannheim, Germany).

### Insulin tolerance test

Insulin tolerance test (ITT) was performed at week 8. After a 4 h fasting, insulin at a dose of 0.6 U/kg^−1^ body weight (Novo Nordisk Pharmaceutical Co., Ltd., China) was injected intraperitoneally. Blood samples were taken from the tail vein at 0, 30, 50, 70, and 90 min after insulin injection, and blood glucose concentrations were measured as described above.

### Blood and tissue sampling

At the end of the study, animals were fasted for 12 h and blood samples were collected by ocular puncture into centrifuge tubes. Serum was isolated by centrifugation at 3,000 g, 4°C for 10 min. Then, all the mice were sacrificed by cervical dislocation with the anesthetic effect of ether. The liver and abdominal adipose tissue were excised and weighed.

### Serum measurements

Fasting serum glucose and insulin levels were measured at the end of the treatment. The serum glucose concentration was determined by the glucose oxidase method (27) with corresponding diagnostic kits (Nanjing Jiancheng Bioengineering Inst., Nanjing, China) according to the instruction manual. Serum insulin level was measured with an ELISA assay kit (CUSABIO BIOTECH Co., Ltd., Hubei, China) with a micro-plate reader (BIO-RAD, Japan). HOMA-IR (28) expressed as an index of insulin resistance was calculated using the homeostasis model assessment: HOMA-IR = fasting glucose (mmol/L)×fasting insulin (*u*U/mL)/22.5.

### Hepatic glucokinase activity and glycogen level determination

The glucokinase activity was measured using a method of spectrophotometric continuous assay (29). One *u*mol glucose-6-phosphate formed per minute per milligram protein was defined as one unit of glucokinase activity. Liver glycogen content was measured using the anthrone-reagent method (30) with corresponding diagnostic kits (Nanjing Jiancheng Bioengineering Inst., Nanjing, China) in accordance with the instruction manual.

### Statistical analysis

All data were evaluated by a one-way analysis of variance along with Duncan's multiple-range test. The data were presented as means±SE. A *P-*value less than 0.05 was considered statistically significant, whereas a *P*-value less than 0.01 was considered very significant.

## Results

### Effect of oat beta-glucan on body weight and abdominal adipose tissue

As indicated in Table 2, there was no significant difference in the body weight among the groups before treatment. During the 10-week experimental period, a significant increase in body weight of MC group was 15.30±0.50 g (53.2% increase) compared with 11.85±1.25 g (40.8% increase) of NC group (*P*<0.05). Mice in both the low-dose and high-dose groups showed a gradual increase in body weight which was significantly less than that of MC group mice. The adipose tissue weight was 1.3 times higher in MC group as compared with that of NC group (*P*<0.05).

**Table 2:** Effect of oat beta-glucan on body weight and abdominal adipose tissue weight

Dietary group	Initial weight (g)	Final weight (g)	Weight gain (g)	Abdominal adipose tissue weight (g)
NC	29.05±0.42	40.90±0.98	11.85±1.25	1.15±0.13
MC	28.75±0.25	44.05±0.35#	15.30±0.50#	1.51±0.07#
PC	28.45±0.32	41.60±0.81	13.15±0.72	1.37±0.07
Low-dose	29.00±0.33	41.10±0.84*	12.10±0.78*	1.31±0.07
High-dose	28.11±0.99	39.80±1.14**	11.69±1.14*	1.25±0.08

NC = normal control; MC = model control; PC = positive control; Low-dose = low-dose control (200 mg/kg^−1^ body weight oat beta-glucan); High-dose = high-dose control (500 mg/kg^−1^ body weight oat beta-glucan). Values are means ± SE (*n*=10).

# *P*<0.05 compared with NC

* *P*<0.05

** *P*<0.01 compared with MC

### Serum glucose, insulin, and insulin resistance index

Fasting serum glucose and insulin levels were 1.3 and 1.2 times higher in MC group than in NC group, respectively. Supplementation of high-dose beta-glucan significantly reduced serum glucose and insulin levels by 15.9 and 14.9% compared with MC group (*P*<0.05). As presented in Table 3, insulin resistance indices calculated by homeostasis model assessment in MC group were significantly higher than in NC group by 1.5 times. However, the insulin resistance was significantly reduced in low-dose and high-dose groups (*P*<0.05 compared with MC group), whereas the high-dose group showed a more significant improvement (*P*<0.01 compared with MC group).

**Table 3:** Effect of oat beta-glucan on serum glucose, insulin, and homeostasis model assessment for insulin resistance (HOMA-IR)

Dietary group	Serum glucose (mmol/L)	Insulin (*u*U/mL)	HOMA-IR
NC	7.07±0.48	6.85±0.71	2.21±0.33
MC	9.27±0.15##	8.05±0.12#	3.32±0.07##
PC	7.45±0.54**	6.59±0.38*	2.20±0.23**
Low-dose	8.02±0.25*	7.76±0.12	2.76±0.82*
High-dose	7.80±0.28*	6.85±0.21*	2.38±0.12**

NC = normal control; MC = model control; PC = positive control; Low-dose = low-dose control (200 mg/kg^−1^ body weight oat beta-glucan); High-dose = high-dose control (500 mg/kg^−1^ body weight oat beta-glucan); Values are means ± SE (*n*=10). HOMA-IR = fasting glucose (mmol/L)×fasting insulin (*u*U/mL)/22.5.

# *P*<0.05

## *P*<0.01 compared with NC

* *P*<0.05

** *P*<0.01 compared with MC.

### Oral glucose tolerance test

**Fig. 1:**
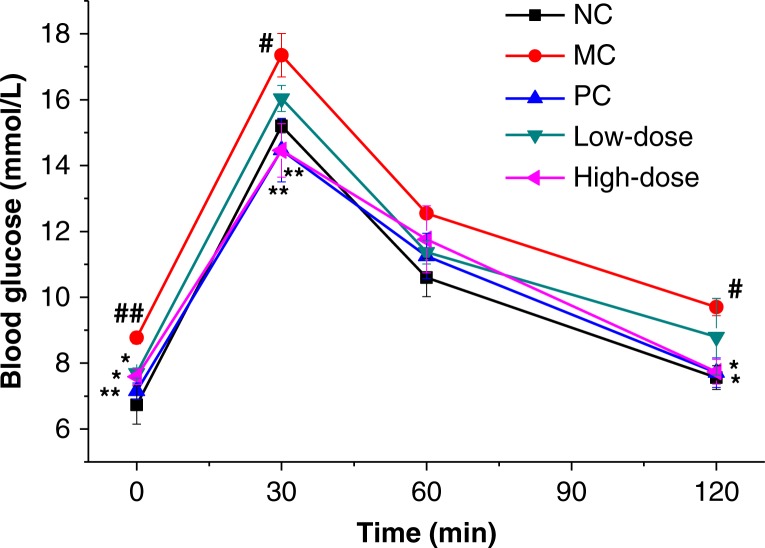
Effect of oat beta-glucan on OGTT blood glucose levels. Values are presented as means±SE (*n*=10 for each group); NC = normal control; MC = model control; PC = positive control; Low-dose = low-dose control (200 mg/kg^−1^ body weight oat beta-glucan); High-dose = high-dose control (500 mg/kg^−1^ body weight oat beta-glucan); #*P*<0.05, ##*P*<0.01 compared with NC; *P<0.05, **P<0.01 compared with MC.

Fig. 1 depicts the effect of beta-glucan on OGTT. Before the glucose load, the blood glucose levels at 0 time were significantly different among groups. Glucose challenge dramatically raised the blood glucose level of MC group compared with NC group at 30 and 120 min indicating glucose intolerance. However, compared with MC group, the high-dose group showed a significant improvement in oral glucose tolerance. Mice in this group had lower blood glucose levels at 0, 30, and 120 min and marginally decreased blood glucose levels at 30 min (*P*<0.01 compared with MC group). The low-dose group exhibited no significant reduction of blood glucose levels at 30, 60, and 120 min intervals except 0 min time point compared with that of MC group.

### Insulin tolerance test

**Fig. 2:**
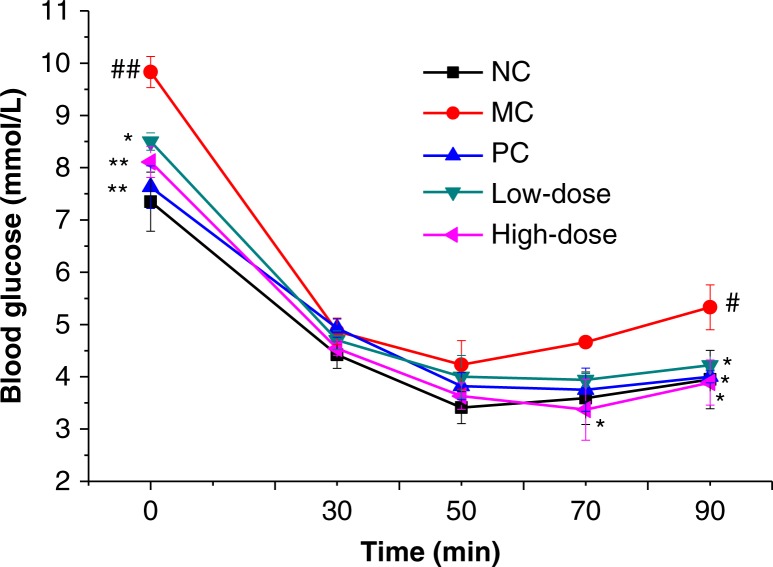
Effect of oat beta-glucan on ITT blood glucose levels. Values are presented as means±SE (*n*=10 for each group); NC = normal control; MC = model control; PC = positive control; Low-dose = low-dose control (200 mg/kg^−1^ body weight oat beta-glucan); High-dose = high-dose control (500 mg/kg^−1^ body weight oat beta-glucan); #*P*<0.05, ##*P*<0.01 compared with NC; *P<0.05, **P<0.01 compared with MC.

After an intraperitoneal injection of insulin, the blood glucose levels of all groups decreased rapidly (Fig. 2). The blood glucose level of MC group at 90 min was significantly higher than that of NC group. No significant difference in glucose concentrations was found among groups at 30 and 50 min intervals. Compared with MC group, only the high-dose group showed a significant decrease in blood glucose levels both at 70 and 90 min time points (*P*<0.05).

### Hepatic glucokinase activity and glycogen content

**Fig. 3:**
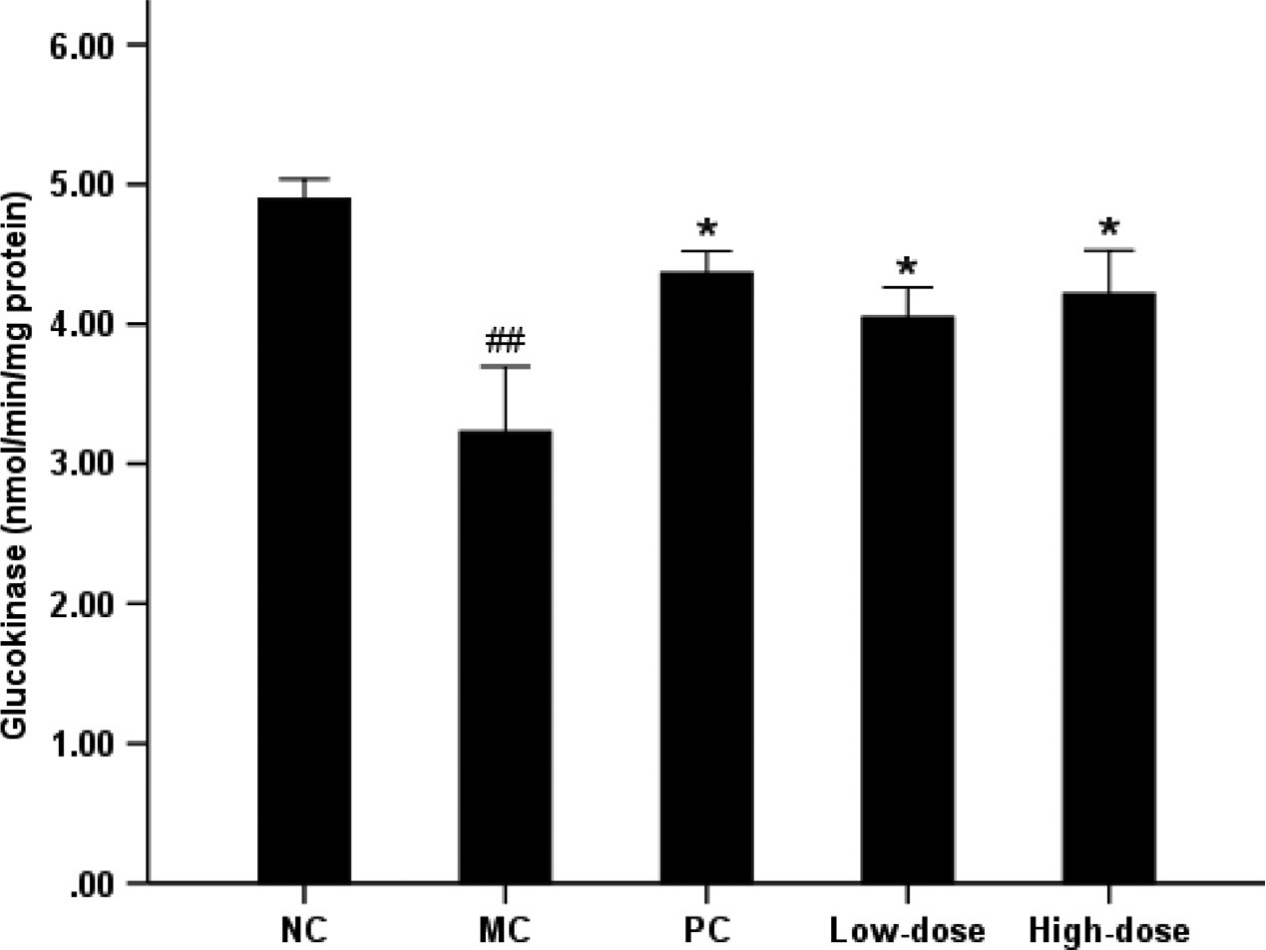
Effect of oat beta-glucan on the hepatic glucokinase. Values are means for 10 mice with SE represented by vertical bars. The mean value was significantly different from that of the NC group at ##*P*<0.01, MC group at *P<0.05, **P<0.01. NC = normal control; MC = model control; PC = positive control; Low-dose = low-dose control (200 mg/kg^−1^ body weight oat beta-glucan); High-dose = high-dose control (500 mg/kg^−1^ body weight oat beta-glucan)

**Fig. 4:**
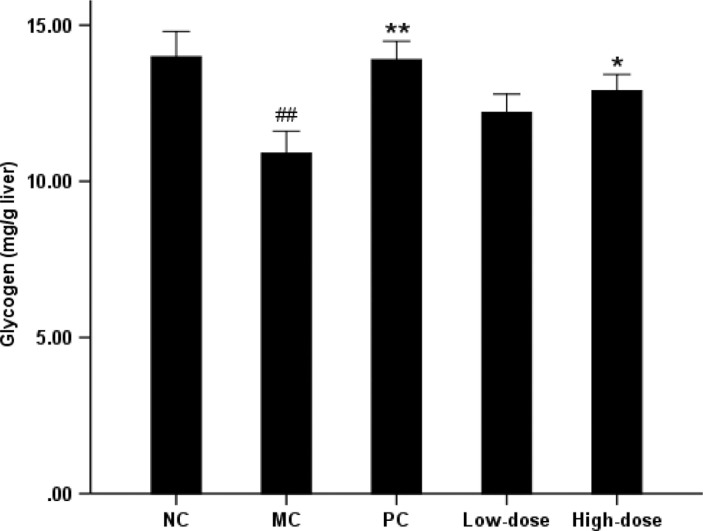
Effect of oat beta-glucan on the hepatic glycogen. Values are means for 10 mice with SE represented by vertical bars. The mean value was significantly different from that of the NC group at ##*P*<0.01, MC group at *P<0.05, **P<0.01. NC = normal control; MC = model control; PC = positive control; Low-dose = low-dose control (200 mg/kg^-1^ body weight oat beta-glucan); High-dose = high-dose control (500 mg/kg^−1^ body weight oat beta-glucan)

The MC group experienced a 33.9% reduction in glucokinase activity compared with NC group (Fig. 3). However, there was elevated hepatic glucokinase activity of 25.4 and 30.7%, respectively, in the low-dose and high-dose groups (*P*<0.05 compared with the MC group). The MC group significantly reduced hepatic glycogen content by 22.0% compared with NC group (Fig. 4). Supplementation of beta-glucan in high-dose group showed a 18.3% (*P*<0.05) increase in hepatic glycogen level, whereas there was no significant difference in low-dose group compared with MC group.

## Discussion

Currently, obesity and type 2 diabetes have become increasingly common throughout the world. It has been shown that obesity caused by increasing the intake of high-calorie food is a major reason for insulin resistance and type 2 diabetes (31). Insulin resistance was more prevalent in obese subjects (6) and increased as weight increased (5). Moreover, the obese mice on high-fat diet exhibited the characters of hyperglycemia besides insulin resistance (32).

This study suggested that high-fat and high-fructose diet combined with fructose drinking water for 10 weeks caused overweight, hyperglycemia, mild hyperinsulinemia, and insulin resistance. The low-dose group did not show significant effect on oral glucose tolerance whereas supplementation with high-dose oat beta-glucan was helpful to maintain glucose homeostasis and clear the postprandial glucose load (Fig. 1). Kim and others (33) reported that higher amount of beta-glucan consumption induced a reduced peak and sustained net increment in the late phase (60 and 120 min) of postprandial glucose response in obese women with increased risk for insulin resistance. Tapola and others (34) reported that oat bran flour high in beta-glucan acted as an active ingredient decreasing postprandial glycemic response of an oral glucose load in subjects with type 2 diabetes. Other studies also showed hypoglycemic effects of beta-glucan in postprandial plasma glucose in control of diabetic subjects (35, 36). Compared with the MC group, the high-dose and low-dose groups showed a significant increase in insulin sensitivity at 70, 80, and 90 min in the ITT, which was one of the first developed methods for the assessment of insulin sensitivity in vivo (37).

As exhibited in Table 2, supplementation of beta-glucan inhibited the subsequent development of obesity which was associated with reduced ability of the body to control blood glucose with normal insulin levels (38). Because increased glucose and insulin concentrations were the primary indicators for insulin resistance and type 2 diabetes (4, 39), beta-glucan markedly improved insulin resistance (Table 3) by decreasing fasting serum glucose and insulin levels both in low-dose and high-dose control groups compared with that of MC group. However, the effect of high-dose beta-glucan in alleviating insulin resistance was more significant and close to that of metformin, a biguanide agent that reduces hyperinsulinemia. It is considered that the improved insulin resistance conferred by beta-glucan contributed to the suppressive effect on high-fat and high-fructose diet which induced impaired glucose tolerance and insulin sensitivity.

In the present study, the activity of hepatic glucokinase, also called hexokinase IV, was reduced by 34% in MC group compared with NC group which was consistent with the observation that high-fat diet reduced hepatic glucokinase in rats (40). Liver glucokinase activity was also markedly suppressed in animal models of diabetes, including the insulin-deficient streptozotocin model and the insulin-resistant Zucker Diabetic Fatty rat (41, 42). Because insulin induced the transcription of glucokinase gene in the liver (43) and regulated glucokinase expression (44), reduced glucokinase activity was generally attributed to defects in insulin secretion or action. Glucokinase was a major component in the glucose-sensing machinery of mammals and played a fundamental role in glucose homeostasis (45, 46). It has been shown that an increased glucokinase level leads to augmented hepatic glycogen storage and a subsequent reduction in glycemia (47). Conversely, removal of glucokinase resulted in reduced hepatic glycogen deposition and hyperglycemia (48). McGarry (49) reported that insulin reduced the output of hepatic glucose by activating glycogen synthesis and glycolysis, and by inhibiting gluconeogesis.

As described above, insulin, glucokinase, and glycogen were closely interrelated with each other. In the present study, both high-dose and low-dose control groups significantly elevated hepatic glucokinase activity, but only the high-dose group had a marked increase in the glycogen level compared with MC group (*P*<0.05). Meanwhile, the high-dose group also exhibited a more significant improvement in insulin resistance (*P*<0.01 compared with MC group), and the effect was dose dependent.

In the high-dose group, the insulin resistance was seemingly mediated by increasing glucokinase activity and reducing hepatic glucose output for liver glycogen deposition to enhance glucose utilization accompanying improvement in oral glucose and insulin tolerance.
